# Exploration of Diffusion Tensor Imaging for Delineating Target Volume Boundary in Glioblastoma Radiotherapy

**DOI:** 10.1111/1754-9485.13873

**Published:** 2025-06-10

**Authors:** Lei Tian, Wenyan Wang, Wei Sun, Huandi Zhou, Zhiqing Xiao, Xuetao Han, Xing Kang, Xiaoying Xue

**Affiliations:** ^1^ Department of Radiotherapy The Second Hospital of Hebei Medical University Shijiazhuang Hebei China; ^2^ Department of Radiotherapy Affiliated Hospital of Hebei University Baoding Hebei China

**Keywords:** ADC, DTI, FA, glioblastoma, radiotherapy, target volume

## Abstract

**Purpose:**

The objective of this study is to investigate the variations in diffusion tensor imaging (DTI) parameters at different distances surrounding the operative cavity, with a specific focus on exploring the potential utility of DTI in accurately delineating radiotherapy clinical target volume for glioblastoma patients.

**Methods:**

A retrospective study was conducted on 41 patients with glioblastoma, in which apparent diffusion coefficient (ADC) and fractional anisotropy (FA) values were measured at various distances beyond the surgical cavity. Recurrent patients were prospectively followed up according to the RANO criteria, aiming to investigate discrepancies between ADC and FA values in recurrent regions compared to normal control tissues prior to recurrence.

**Results:**

The rADC and rFA ratio approach 1 at a distance of 3 cm beyond the cavity. At the edge of the operative cavity and 2 cm beyond, the subtotal resection (STR) group exhibited higher ADC and rADC values compared to the gross total resection (GTR) group (*p* < 0.05). Similarly, FA and rFA values in the STR group were lower than those in the total resection group both at 1 cm beyond and 2 cm beyond (*p* < 0.05). Conventional MRI did not reveal any abnormalities prior to marginal or distant recurrence; however, the ADC value within this region was higher than that of control normal tissues (*p* = 0.023).

**Conclusions:**

The margins of GBM tumour invasion are typically not isotropic and could be > 2 cm and sometimes up to 3 cm. We recommend appropriately larger expansion of the target volume for patients with subtotal tumour resection. The utilisation of DTI in delineating the boundary of GBM's radiotherapy clinical target volume represents a promising avenue that holds potential to enhance precision and accuracy.

## 1 Introduction

Glioblastoma Multiforme (GBM) is the most prevalent primary malignant intracranial tumour, resulting in a 5‐year survival rate of less than 10% [1]. The treatment of maximal surgical resection combined with chemoradiotherapy has remained stagnant over the past two decades [2, 3, 4]. Despite the emergence of Tumour Treating Fields (TTF), which has improved median GBM survival from 16 to 20.9 months, its limited availability and high cost restrict its applicability to only a fraction of patients [5]. Therefore, enhancing the therapeutic efficacy in GBM patients holds significant clinical importance. However, achieving complete excision through surgery is hindered by the highly invasive growth pattern of GBM and functional constraints imposed by tumour location, leading to frequent postoperative recurrences in patients with GBM. Radiotherapy, a non‐invasive local treatment modality, effectively eradicates residual tumours and subclinical lesions surrounding the surgical cavity following surgery. It serves as a crucial adjuvant therapy in the postoperative management of GBM [6]. Given the absence of breakthroughs in chemotherapy effectiveness or targeted/immunotherapy approaches, optimising radiotherapy outcomes becomes particularly crucial as it currently represents the most practical and accessible means for improving GBM patient survival.

The conventional method of target volume delineation based on preoperative and pre‐radiotherapy conventional MRI has evident limitations, such as imprecise lesion identification and the challenge of differentiating between vasogenic and tumour‐related edema [7, 8]. Therefore, the exploration of imaging techniques that can objectively depict the characteristics of GBM tumours and infiltration areas, as well as individualised outlining of clinical target volume, is crucial to ensure the effectiveness of radiotherapy. Multimodal imaging techniques based on functional magnetic resonance images have been employed in diagnosing, differentiating, and predicting prognosis in GBM patients [9, 10]. GBMs exhibit infiltrative growth patterns with preferential infiltration along white matter fibre bundles rather than grey matter regions [11]. Diffusion Tensor Imaging (DTI) is an imaging technique that characterises the diffusion of water molecules in a specific direction based on Diffusion‐weighted Imaging (DWI). It can be utilised to detect tumour infiltration through the apparent diffusion coefficient (ADC) [12] and non‐invasively visualise cerebral white matter fibre bundles through anisotropy measurements such as fractional anisotropy (FA) [12]. Therefore, DTI holds potential for determining glioma infiltration trajectory and extent, as well as identifying tumour boundaries. However, there are limited studies guiding their clinical application. Hence, this study aims to analyse changes in ADC and FA values at various distances surrounding the GBM operative cavity and explore the significance of DTI in delineating clinical target volume for GBM radiotherapy. The findings will provide valuable references and theoretical foundations for practical clinical work.

## 2 Materials and Methods

### 2.1 General Information

Retrospectively selected patients with GBM who underwent surgery and received the standard STUPP regimen at the Second Hospital of Hebei Medical University between August 2017 and June 2022 were included in this study. The target volumes for all patients were delineated in accordance with the RTOG standards [8]. During radiotherapy, concurrent temozolomide chemotherapy was administered, followed by 6 months of temozolomide maintenance chemotherapy. Prior to radiotherapy, all patients completed MRI scans of the head, including T1WI, T2WI/Fliar, T1‐weighted, and DTI. The postoperative residuals were determined based on preoperative, postoperative, and pre‐radiotherapy MRI scans by a Radiologist and another Radiation Oncologist. Patient demographics and molecular pathology markers were collected.

### 2.2 Determination of Tumour Recurrence and Definition of Recurrence Pattern

The determination was primarily based on the Response Assessment in Neuro‐Oncology (RANO) criteria [13]. The determination of recurrence can also be achieved through multidisciplinary team (MDT) discussion involving neurosurgeons, radiation oncologists, radiologists, and pathologists.

The radiotherapy localisation CT and treatment planning dose curves were fused with the MRI images at the time of the first recurrence through the MIM image workstation, and the recurrent lesion was outlined based on the recurrent MRI, and the percentage of the volume of the recurrent lesion within the 95% prescription dose (60Gy) isodose line was calculated. More than 80% was defined as In‐field.

Recurrence (In‐field), 20%–80% for Marginal recurrence (Marginal), and less than 20% for Outside recurrence (Distant) [14].

### 2.3 DTI Data Processing and Measurement

All MRI sequences were acquired using a Philips 3.0 T MRI scanner. Initially, conventional T1WI and T2WI sequences were performed, followed by DTI using anatomical positioning lines consistent with those used in conventional MRI. The acquisition parameters for the DTI scans were as follows: a single‐shot echo‐planar imaging (EPI) sequence based on the spin‐echo (SE) technique, 13 diffusion directions, repetition time (TR) 2776 ms, echo time (TE) 60 ms, matrix size 128 × 128, field of view (FOV) 224 mm, number of excitations (NEX) 1, and b‐values of 0 and 1000 s/mm^2^. The DTI raw images were imported into the Philips MR post‐processing workstation for subsequent processing. The workstation was utilised to generate ADC and FA maps, from which regions of interest (ROIs) were selected with an area of 20 ± 2 mm^2^, avoiding evident cystic tissues, haemorrhage, necrosis, calcification, and anatomical barriers. Subsequently, the ADC values and FA values within these ROIs were quantified.

**FIGURE 1:**
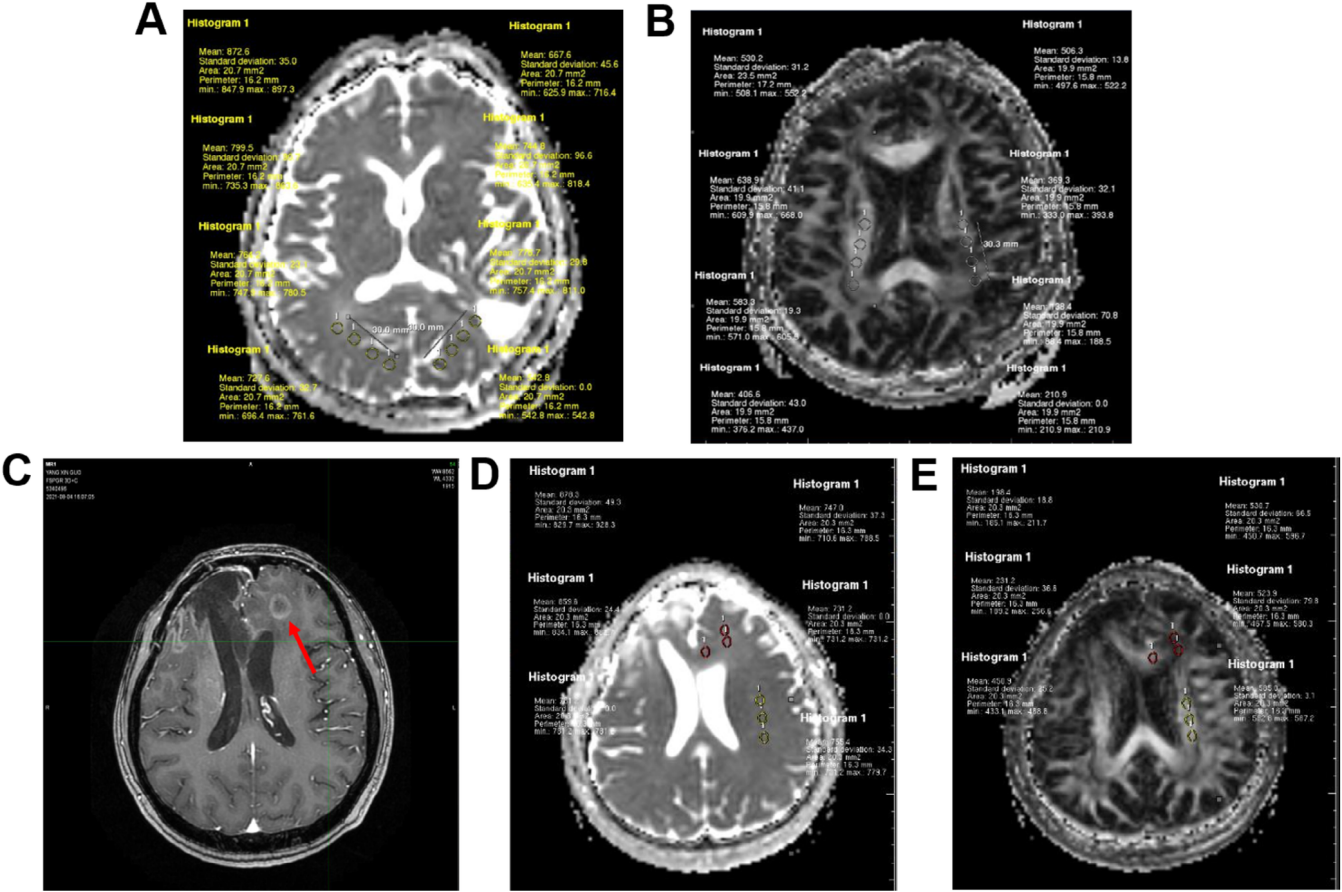
ROIs selection and measurement. Illustration of the selection and measurement of ROIs at varying distances surrounding the surgical cavity. The small words on the figure are the actual measurements. (A) ADC map; (B) FA map. Illustration of ROIs selection and measurement for recurrent cases. The red circles indicate areas of recurrent tumours, while the yellow circles represent control normal tissue. (C) T1‐enhanced MRI images after recurrence; (D) ADC map prior to recurrence; (E) FA map prior to recurrence.

Randomly select three operative cavities from the axial ADC and FA maps. Subsequently, randomly select a ROI at the margin of each operative cavity (if residual tumour is present, select the margin of the enhanced lesion) to obtain a total of three ROIs. In ADC maps, each ROI was uniformly expanded by 1 cm to form ROI_1cm_, 2 cm for ROI_2cm_, and 3 cm for ROI_3cm_. Similarly, in FA maps, each ROI was expanded by 1 cm along the direction of white matter fibre tracts to create ROI_1cm_, 2 cm for ROI_2cm_, and 3 cm for ROI_3cm_. A control reference point of equal size was selected from corresponding normal brain tissue on the contralateral side of each respective ROI. In cases where no normal tissue existed on this side (e.g., within an operative cavity), a control reference point from normal brain tissue on the same side as that particular ROI was chosen instead (Figure 1A,B).

Based on post‐recurrence MRI images, the corresponding areas of recurrence were identified in the ADC map and FA map prior to radiotherapy. Three randomly selected regions of interest (ROIs) were chosen with the same method as before. A similarly sized ROI was also selected in the contralateral normal brain tissue for each ROI (if no normal tissue was available on the contralateral side due to factors such as operative cavity, a control reference point was chosen in the ipsilateral normal brain tissue) (Figure 1C–E). The average values of ADC and FA were measured across all ROIs.

### 2.4 Numerical Processing and Data Analysis

The ADC and FA values within each ROI were recorded and converted to standard units by dividing them by 1000. The ADC and FA values from the ROIs in the three different axial images are averaged to obtain ADC_0cm_, ADC_1cm_, ADC_2cm_, ADC_3cm_, FA_0cm_, FA_1cm_, FA_2cm_, and FA_3cm_. Given that the standard thresholds for ADC and FA values vary across different anatomical locations, a direct comparison of these values lacks comprehensiveness. Comparing the ratios of ADC and FA values to those of the corresponding normal contralateral control tissue can provide a more objective reflection of abnormalities at various locations, thereby yielding rADC_0cm_, rADC_1cm_, rADC_2cm_, rADC_3cm_, rFA_0cm_, rFA_1cm_, rFA_2cm_, and rFA_3cm_.

Analyse the trends of ADC and FA values and ratios in the 0, 1, 2 and 3 cm regions adjacent to the surgical cavity; examine the trends of ADC and FA values and ratios in these regions for different molecular typing as well as different Ki‐67 index groups. Analyse the disparity in ADC and FA values between recurrent areas prior to recurrence compared to normal control tissue; investigate the overall recurrence pattern along with the impact of various clinical factors on recurrence patterns.

### 2.5 Statistical Analysis

Statistical analysis encompassed a range of methodologies, including the Shapiro–Wilk normality test to assess normal distribution, independent samples and paired samples *t*‐tests, Mann–Whitney *U* test for non‐normally distributed two‐group comparisons, and Kruskal‐Wallis test for non‐normally distributed multiple‐group comparisons. The differences in composition ratios between different groups were evaluated using chi‐square test and Fisher exact test. A significance level of *p* < 0.05 was considered statistically significant. Statistical analysis and visualisation were conducted using R version 3.6.3 (http://www.r‐project.org/), employing the “stats” package, “car” package, and “cmprsk” package for statistical analysis as well as the “ggplot2” package for visualisation.

## 3 Results

### 3.1 General Information

This study enrolled a total of 41 patients, comprising 22 males and 19 females, with an average age of 55 ± 11.7 years. Among these, 24 cases underwent complete resection and were included in the gross total resection group, whereas 17 cases underwent subtotal resection and were included in the STR group. MGMT promoter methylation status was observed in 32 patients. Similarly, TERT mutation status was identified in another set of 32 patients. Notably, among these patients, a total of 32 individuals had available information on both MGMT promoter methylation and TERT mutation. The median value for Ki‐67 was determined to be 30% (Table 1).

**TABLE 1:** General information.

Characteristic	Levels	Overall
*n*		41
Age		55 ± 11.7
Gender		*n* = 41
	Female	19 (46.3%)
	Male	22 (53.7%)
Resection		*n* = 41
	STR	17 (41.5%)
	GTR	24 (58.5%)
MGMT promoter		*n* = 32
	Methylation	14 (43.8%)
	Unmethylation	18 (56.2%)
TERT promoter		*n* = 32
	Mutant	22 (68.8%)
	Wild‐type	10 (31.2%)
MGMT/TERT		*n* = 31
	UmMGMT & TERT‐mt	14 (45.2%)
	Else	17 (54.8)
Ki‐67 (%)		30 (20.50)

*Note:* IDH‐mutant GBM have been excluded from the original classification according to the new 2021 (5th) WHO classification of CNS tumours [15].

### 3.2 Variation Trends of DTI Parameters at Different Distances From the Surgical Cavity

**FIGURE 2:**
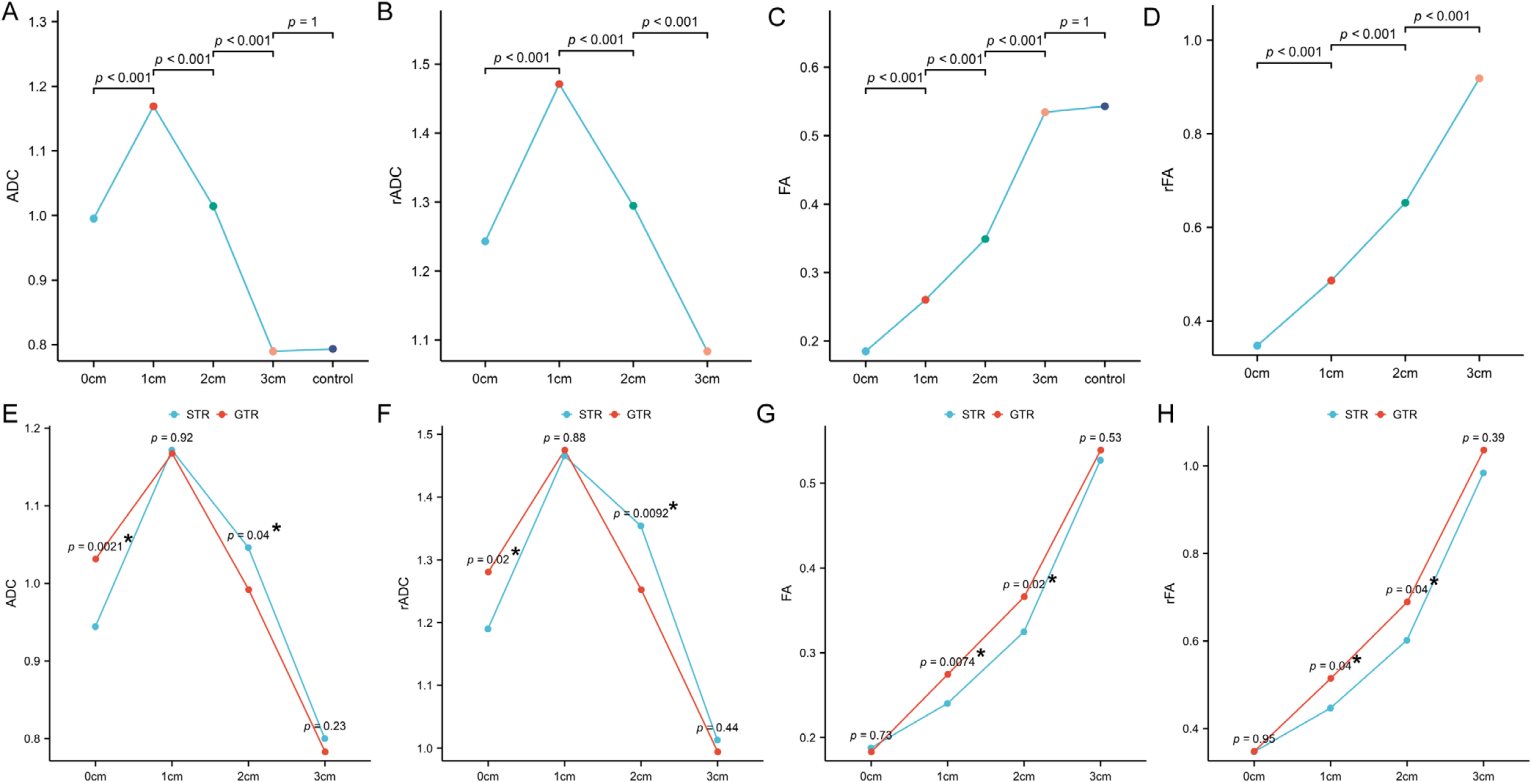
Variation of DTI and DWI parameters at different distances from the margin of the surgical cavity. (A) ADC, (B) rADC, (C) FA, (D) rFA. STR vs. GTR: (E) ADC, (F) rADC, (G) FA, (H) rFA. Statistical significance was denoted by *.

The ADC value and rADC ratio exhibited a trend of initially increasing and then decreasing with distance, peaking at 1 cm outside the surgical cavity. Statistically significant differences were observed at different distances. There was no difference between ADC_3cm_ and the control group, while rADC_3cm_ was close to 1 (Figure 2A,B). Similarly, FA value and rFA ratio showed a gradual increase with distance, with statistically significant differences observed at different distances. There was no difference between FA_3cm_ and the control group, while rFA_3cm_ was close to 1 (Figure 2C,D).

The ADC and rADC values of the STR group were significantly higher than those of the Gross total resection (GTR) group at both the edge of the operative cavity and 2 cm outside, as depicted in Figure 2E,F. Moreover, at distances of 1 and 2 cm outside the operative cavity, the FA and rFA values of the STR group were significantly lower compared to those of the GTR group, as shown in Figure 2G,H. These results indicate that patients with subtotal resection have more tumour infiltration in the corresponding location.

### 3.3 Variation Trends of DTI Parameters at Different Distances From the Surgical Cavity in Relation to Different Ki‐67 Indices

**FIGURE 3:**
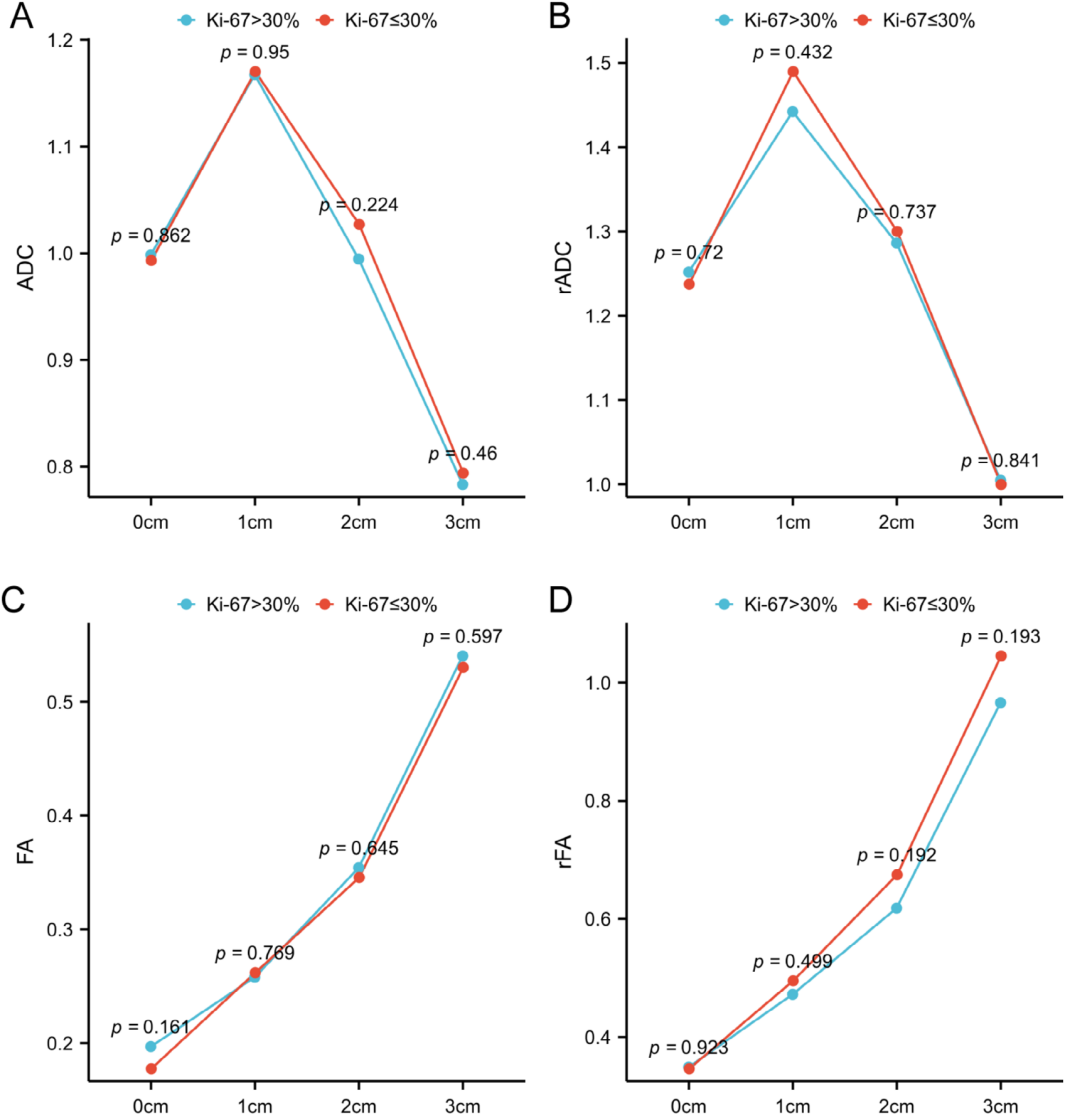
Variation of DTI and DWI parameters at different distances from the margin of the surgical cavity in relation to different Ki‐67 indices. (A) ADC; (B) rADC; (C) FA; (D) rFA. ns, no statistical significance.

The Ki‐67 index was analysed by stratifying the entire patient cohort into high and low groups based on a median value of 30%. It was observed that both ADC and rADC exhibited a trend of initially increasing and then decreasing with different Ki‐67 indices (Figure 3A,B). Similarly, FA and rFA demonstrated a gradual increasing trend without statistically significant intergroup variations (Figure 3C,D).

### 3.4 Variation Trends of DTI Parameters at Different Distances From the Surgical Cavity for Distinct Molecular Fractions

**FIGURE 4:**
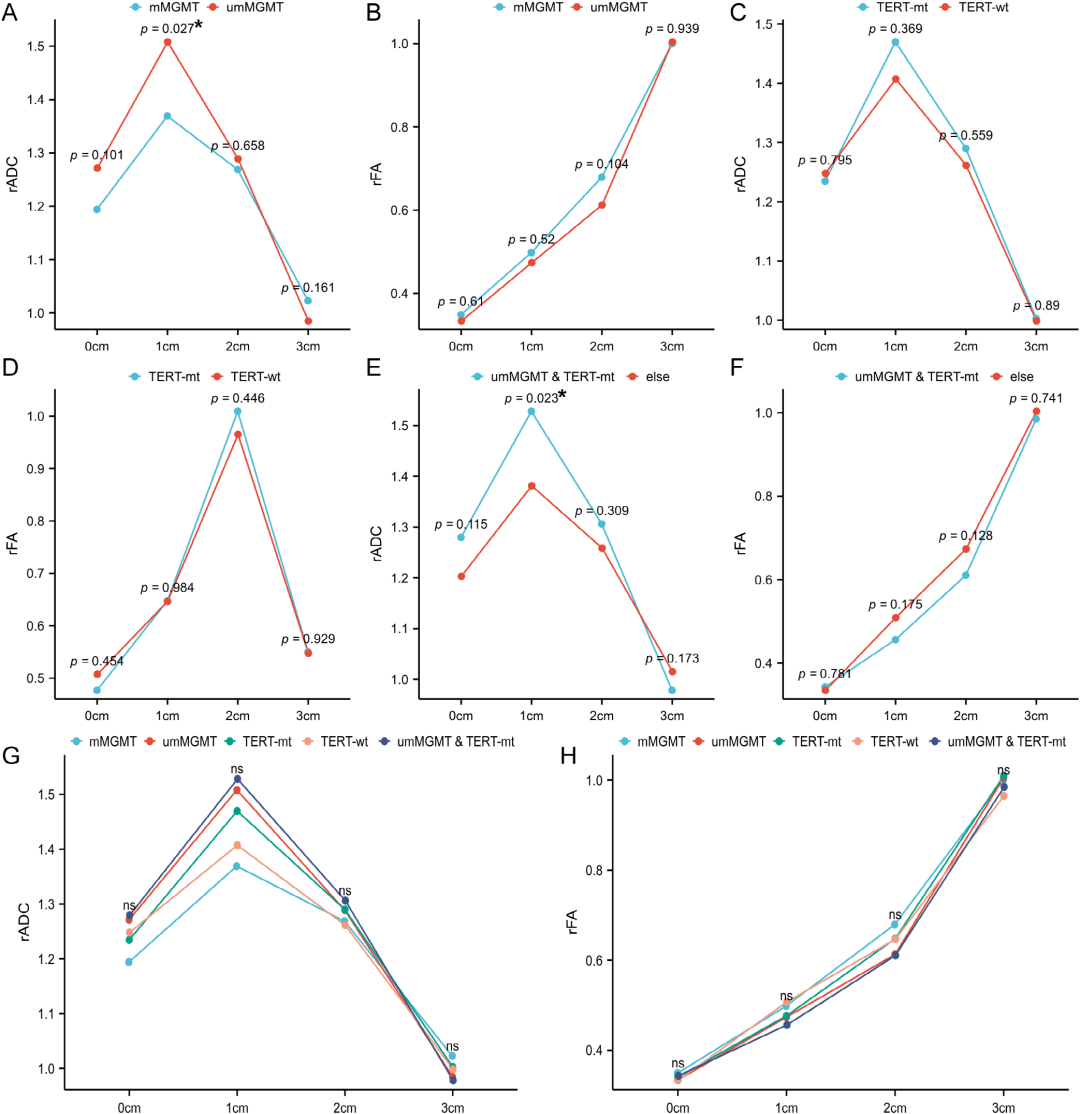
Variation of DTI and DWI parameters at different distances from the margin of the surgical cavity for distinct molecular fractions. (A) MGMT‐rADC; (B) MGMT‐rFA; (C) TERT‐rADC; (D) TERT‐rFA; (E) MGMT/TERT‐rADC; (F) MGMT/TERT‐rFA; (G) ALL‐rADC; (H) ALL‐rFA. ns, no statistical significance. *, statistical significance.

The ADC and rADC values of patients with different molecular subtypes exhibited a biphasic trend, initially increasing and then decreasing. At 1 cm, the rADC values were significantly higher in the MGMT promoter non‐methylated group compared to the methylated group (*p* = 0.027, Figure 4A). Furthermore, the rADC values were significantly higher in the MGMT unmethylation combined with TERT mutation group compared to other groups at 1 cm (*p* = 0.023, Figure 4E), while no statistically significant differences were observed for other comparisons (Figure 4B–DF). Additionally, there was no statistical difference in FA and rFA values among all groups (Figure 4G,H).

### 3.5 Recurrence Analysis

**FIGURE 5:**
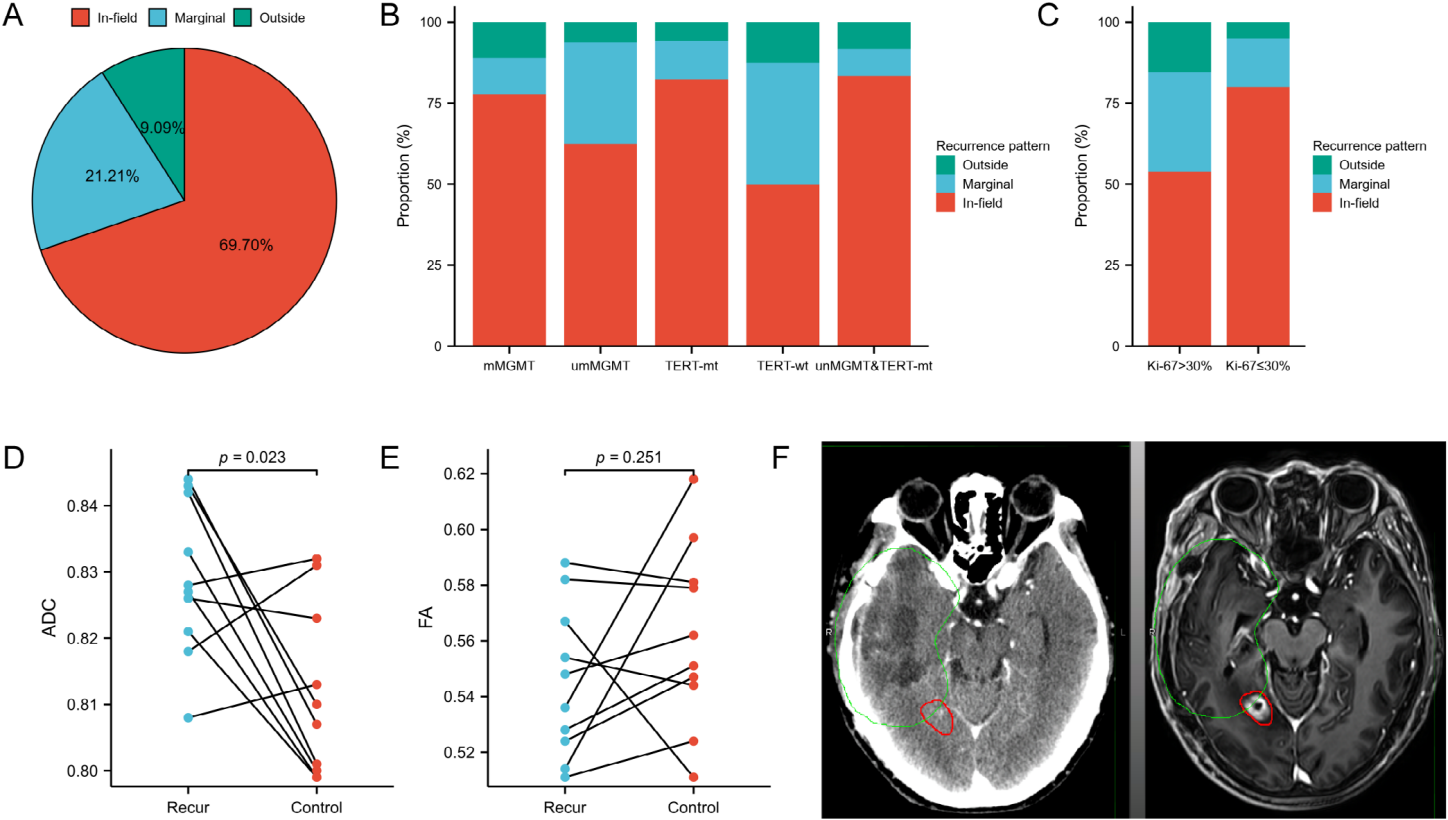
The analysis of recurrence patterns: (A) overall patient population; (B) patients with distinct molecular characteristics, (C) patients stratified by different Ki‐67 index levels. Comparison of DTI parameters in recurrent regions between patients with Marginal and Outside patterns and normal tissue prior to recurrence: (D) ADC values; (E) FA values; (F) An illustrative example of marginal recurrence: MRI after recurrence was fused with the initial CT scan from radiotherapy, where the green circle represents the initial radiotherapy target volume and the red circle indicates the location of recurrence.

A total of 33 patients with recurrence were included in our study, comprising 23 (69.7%) cases of local recurrence, 7 (21.21%) cases of margin recurrence, and 3 (9.09%) cases of distant recurrence (Figure 5A). Among different molecular subtypes, patients with non‐methylated MGMT promoter and wild‐type TERT promoter exhibited a relatively higher proportion of margin recurrence (Figure 5B). Additionally, patients with Ki‐67 > 30% showed a relatively higher percentage of margin recurrence (Figure 5C).

In consideration of the fact that in‐field recurrence often occurs within the operative cavity, where it is challenging to measure meaningful DTI parameters, patients with marginal and distant recurrence were included in this study. It was observed that the ADC value of the pre‐recurrence area in these patients was significantly higher than that of normal tissues (*p* = 0.023) (Figure 5D). Additionally, there was a tendency for a reduction in FA values compared to normal tissues; however, this difference did not reach statistical significance (*p* = 0.251) (Figure 5E). These findings suggest that DTI parameters possess certain early warning capabilities for recurrence and can show the tumour boundary more objectively and guide the delineation of clinical target volume (Figure 5F).

## 4 Discussion

Controversy has persistently surrounded the recommended delineation of target areas for GBM radiotherapy in authoritative guidelines, yet a definitive solution remains elusive. Presently, both EORTC and ASTRO guidelines advocate referencing traditional plain and enhanced MRI for target delineation; however, several limitations exist: (1) T1 enhancement cannot objectively depict the true tumour area due to variations in patient population and lesion characteristics [16]; (2) GBM exhibits significant heterogeneity in infiltrative and invasive growth patterns attributed to clinical pathological factors; thus uniform expansion may result in target area leakage and unnecessary enlargement [17]; (3) Discriminating between ordinary vascular‐origin edema and abnormal signals associated with tumour infiltration based solely on T2/Flair imaging proves challenging. Delineating CTV solely based on this criterion may lead to excessive target area size causing unwarranted radiation damage while also impacting dose escalation [18]. With the advancement of advanced imaging technology, DTI emerges as a novel functional examination method developed from DWI by employing multiple linear diffusion‐sensitive gradients. Comparative studies between DTI images and conventional postoperative MRI‐T2/Flair abnormal high signal areas in GBM patients have revealed discrepancies in identifying tumour tissue edges but also highlighted their complementary nature; thus implying that incorporating DTI can enhance postoperative radiotherapy target delineation for gliomas [19]. Fei et al. [20] conducted a retrospective analysis on the prognostic outcomes of patients with glioma who underwent radiation therapy target delineation using multimodal MRI techniques, including DTI and PWI, compared to conventional MRI. In the control cohort, the GTV was delineated using axial T1‐weighted contrast‐enhanced images. The GTV was expanded 1–2 cm (CTV) for Grade III and up to 2–3 cm (CTV) for grade IV and received 60 Gy/30 fx. In the study cohort, CTV1 was defined as the resection cavity and abnormal regions identified on DTI and PWI, with an additional 5 mm margin. CTV2 encompassed CTV1 plus an additional 5 mm margin, including the ependyma. CTV1 was administered a dose of 60 Gy/30 fx, while the dose for CTV2 remains unspecified. The study revealed that patients whose target volume was delineated using multimodal MRI had a survival advantage, which was also identified as an independent prognostic factor. These findings suggest that exploring the use of DTI and other multimodal MRI techniques for glioma target delineation is warranted; however, specific methods and clear quantitative standards for implementing this approach in clinical practice need further development.

In this study, the measurement of ADC values was conducted uniformly expanding outward in all directions to investigate its distribution since ADC reflects water molecule diffusion without directionality. However, due to the directional characteristics along white matter fibre bundles exhibited by FA values, a more accurate approach involved measuring FA values by expanding outward along the direction of fibre bundles at the edge to better align with glioma growth patterns. This measurement method may serve as a reference for revising target delineation in clinical applications. To account for individual differences and anatomical variations depending on tumour location that can affect actual measurements of ADC and FA, rADC and rFA ratios were calculated for further research analysis to objectively reflect abnormal conditions within the study area. Both ADC and FA parameters deviate from those observed in normal tissue beyond 2 cm outside the surgical cavity but converge towards similarity with normal tissue at 3 cm. These findings suggest that GBM infiltrates an area extending no less than 2 cm beyond its visible boundaries; therefore, radiation therapy target expansion for GBM patients should be no less than 2 cm. Currently recommended guidelines by NCCN as well as European guidelines propose target volume expansions smaller than 2 cm based on certain literature [21, 22]. However, considering our study results, caution should still be exercised regarding this practice. Further grouping analysis revealed significant differences in ADC and FA parameters at 2 cm between the STR group and the GTR group. This suggests that postoperative residual GBM patients exhibit a broader spectrum of tumour infiltration, indicating that expanding the radiation target area by 2 cm may not be sufficient and necessitates a larger margin. However, this study did not further classify or investigate within the range of 2–3 cm, thus leaving uncertainty regarding the boundary for CTV expansion in patients with subtotal resection. Further refinement of research is warranted to ascertain this.

However, there is no statistically significant difference between different molecular subtypes and high/low Ki‐67 index groups. Nevertheless, patients with MGMT promoter non‐methylation combined with TERT promoter mutations, as well as those with MGMT promoter non‐methylation alone, demonstrate higher rADC ratios and lower rFA ratios across various ranges outside the surgical cavity. This suggests that tumours of these molecular subtypes have a greater extent of infiltration, necessitating a wider expansion of the radiation target area. This finding aligns with the recognised clinical phenotype of glioblastoma characterised by high invasiveness and poor prognosis in cases with these molecular subtypes. Subsequent analysis of recurrence patterns reveals a higher proportion of marginal recurrences among patients with MGMT promoter non‐methylation and high Ki‐67 index, further indicating that conventional expansion methods for target areas are inadequate for this patient population, requiring an expanded range. Despite the lack of statistical significance and the discrepancy with the findings of the current study [23], these findings suggest that exploring individualised precise delineation of target areas based on GBM's molecular features is worth considering as a research direction. The lack of statistical differences may be attributed to the small sample size in this study and potential measurement errors.

In the final recurrence analysis, we observed that over 90% of recurrences were either local or marginal, suggesting that the majority of recurrences occurred within the previously irradiated region. This finding indicates limited value in refining the target boundary. Conversely, a more precise delineation of the target volume could prevent unnecessary over‐irradiation and facilitate the development of dose escalation trials, which is a key objective of our study. The recurrence analysis revealed significantly higher ADC values in these areas compared to contralateral normal tissues, indicating abnormal changes in ADC parameters before recurrence. Interestingly, conventional MRI did not detect any significant abnormalities in the recurrent area prior to radiotherapy. By identifying abnormal ADC parameters, we can effectively avoid missing irradiation targets and enhance the efficacy of radiotherapy. Notably, there is limited research available on this aspect; however, our preliminary findings highlight the potential application value of DTI for delineating radiation target boundaries during glioma treatment. It should be noted that no significant results were obtained for FA values, possibly due to anatomical limitations imposed by neural fibre bundle locations; further investigation is warranted.

In summary, the DTI parameters of GBM patients still differ from normal tissue at a distance of 2 cm outside the surgical cavity, but become similar to normal tissue at 3 cm. This suggests that the margins of GBM tumour invasion are typically not isotropic and could be > 2 cm and sometimes up to 3 cm. The DTI parameters in patients with subtotal resection show significant differences compared to those with gross total resection at a distance of 2 cm outside the surgical cavity, indicating a need for larger expansion in postoperative residual GBM patients beyond 2 cm. Abnormal changes in ADC parameters occur prior to recurrence in recurrent areas, which can guide determination of radiation therapy target boundary. The limited sample size of this study, along with the nerve fibre damage induced by the surgery and variations in surgical techniques, may influence the research outcomes. Future studies should aim to increase the sample size and employ preoperative DTI to mitigate the confounding effects of surgery, thereby facilitating a more precise exploration of the relationship between FA values and tumour invasion. This approach will enable the design of more robust clinical trials to yield better outcomes. Moreover, other advanced imaging modalities, such as other fMRI and fluorine‐18‐fluoroethyltyrosine positron emission tomography (FET‐PET), offer distinct advantages in guiding target volume delineation and represent promising avenues for future investigation. Nonetheless, this study has provided preliminary evidence supporting DTI parameters as quantifiable indicators for precise determination of individualised GBM radiotherapy clinical target volume boundaries—a promising avenue worth exploring that could optimise target delineation patterns, improve targeting accuracy, and inform individualised adjustment of radiation dose.

## Ethics Statement

The study was approved by the Ethics Committee of the Second Hospital of Hebei Medical University and conducted in accordance with the ethical standards specified in the Helsinki Declaration (2013 revision in Brazil).

## Conflicts of Interest

The authors declare no conflicts of interest.

## Data Availability

The data that support the findings of this study are available on request from the corresponding author. The data are not publicly available due to privacy or ethical restrictions.
